# Sex differences in COVID-19 mortality risk in patients on kidney function replacement therapy

**DOI:** 10.1038/s41598-022-22657-4

**Published:** 2022-10-26

**Authors:** Priya Vart, Raphaël Duivenvoorden, Aaltje Adema, Adrian Covic, Patrik Finne, Nicole Heijtink-ter Braak, Kaisa Laine, Marlies Noordzij, Marcel Schouten, Kitty J. Jager, Ron T. Gansevoort, ERACODA Collaborators, Jeroen B. van der Net, Marie Essig, Peggy W. G. du Buf-Vereijken, Betty van Ginneken, Nanda Maas, Brigit C. van Jaarsveld, Frederike J. Bemelman, Farah Klingenberg-Salahova, Frederiek Heenan-Vos, Marc G. Vervloet, Azam Nurmohamed, Liffert Vogt, Daniel Abramowicz, Sabine Verhofstede, Omar Maoujoud, Thomas Malfait, Jana Fialova, Edoardo Melilli, Alexandre Favà, Josep M. Cruzado, Nuria Montero Perez, Joy Lips, Harmen Krepel, Harun Adilovic, Daniela Radulescu, Maaike Hengst, Constantijn Konings, Andrzej Rydzewski, Philippe Braconnier, Daniel Weis, Ryszard Gellert, João Oliveira, Daniela G. Alferes, Elena V. Zakharova, Patrice Max Ambühl, Rebecca Guidotti, Andrea Walker, Fanny Lepeytre, Clémentine Rabaté, Guy Rostoker, Sofia Marques, Tijana Azasevac, Gordana Strazmester Majstorovic, Dajana Katicic, Marc ten Dam, Thilo Krüger, Szymon Brzosko, Vassilios Liakopoulos, Adriaan L. Zanen, Susan J. J. Logtenberg, Lutz Fricke, Olexandr Kuryata, Jeroen J. P. Slebe, Samar Abd ElHafeez, Delphine Kemlin, Jacqueline van de Wetering, Marlies E. J. Reinders, Dennis A. Hesselink, J. Kal-van Gestel, Jaromir Eiselt, Lukas Kielberger, Hala S. El-Wakil, Martine Verhoeven, Ian Logan, Cristina Canal, Carme Facundo, Ana M. Ramos, Alicja Debska-Slizien, Nicoline M. H. Veldhuizen, Eirini Tigka, Maria Anna Polyzou Konsta, Stylianos Panagoutsos, Francesca Mallamaci, Adele Postorino, Francesco Cambareri, Irina Matceac, Ionut Nistor, J. H. M. Groeneveld, Jolanda Jousma, Marjolijn van Buren, Fritz Diekmann, Federico Oppenheimer, Miquel Blasco, Tiago Assis Pereira, Augusto Cesar S. Santos, Carlos Arias-Cabrales, Marta Crespo, Laura Llinàs-Mallol, Anna Buxeda, Carla Burballa Tàrrega, Dolores Redondo-Pachon, Maria Dolores Arenas Jimenez, Alberto Mendoza-Valderrey, Ana Cristina Martins, Catarina Mateus, Goncalo Alvila, Ivo Laranjinha, Julia M. Hofstra, Machiel A. Siezenga, Antonio Franco, David Arroyo, Sandra Castellano, Maria Luisa Rodríguez-Ferrero, Sagrario Balda Manzanos, R. Haridian Sosa Barrios, Wim Lemahieu, Karlijn Bartelet, Ahmet Burak Dirim, Erol Demir, Mehmet Sukru Sever, Aydin Turkmen, Seda Şafak, Daan A. M. J. Hollander, Stefan Büttner, Aiko P. J. de Vries, Soufian Meziyerh, Danny van der Helm, Marko Mallat, Hanneke Bouwsma, Sivakumar Sridharan, Kristina Petruliene, Sharon-Rose Maloney, Iris Verberk, Frank M. van der Sande, Maarten H. L. Christiaans, Marc H. Hemmelder, N. MohanKumar, Marina Di Luca, Serhan Z. Tuğlular, Andrea B. Kramer, Charles Beerenhout, Peter T. Luik, Julia Kerschbaum, Martin Tiefenthaler, Bruno Watschinger, Vadim A. Stepanov, Alexey B. Zulkarnaev, Kultigin Turkmen, Ilaria Gandolfini, Umberto Maggiore, Anselm Fliedner, Anders Åsberg, Geir Mjoen, Hitoshi Miyasato, Carola W. H. de Fijter, Nicola Mongera, Stefano Pini, Consuelo de Biase, Angele Kerckhoffs, Anne Els van de Logt, Rutger Maas, Luuk B. Hilbrands, Olga Lebedeva, Veronica Lopez, Louis J. M. Reichert, Jacobien Verhave, Denis Titov, Ekaterina V. Parshina, Luca Zanoli, Carmelita Marcantoni, Gijs van Kempen, Liesbeth E. A. van Gils-Verrij, John C. Harty, Marleen Meurs, Marek Myslak, Yuri Battaglia, Paolo Lentini, Edwin den Deurwaarder, Maria Stendahl, Hormat Rahimzadeh, Ivan Rychlik, Carlos J. Cabezas-Reina, Ana Maria Roca, Ferdau Nauta, İdris Sahin, Eric Goffin, Nada Kanaan, Laura Labriola, Arnaud Devresse, Anabel Diaz-Mareque, Armando Coca, Gabriel de Arriba, Björn K. I. Meijers, Maarten Naesens, Dirk Kuypers, Bruno Desschans, Annelies Tonnerlier, Karl M. Wissing, Ivana Dedinska, Giuseppina Pessolano, Shafi Malik, Evangelia Dounousi, Evangelos Papachristou, Stefan P. Berger, Jan Stephan F. Sanders, Casper F. M. Franssen, Akin Özyilmaz, Jadranka Buturović Ponikvar, Andreja Marn Pernat, Damjan Kovac, Miha Arnol, Robert Ekart, Alferso C. Abrahams, Femke M. Molenaar, Arjan D. van Zuilen, Sabine C. A. Meijvis, Helma Dolmans, Ekamol Tantisattamo, Pasquale Esposito, Jean-Marie Krzesinski, Jean Damacène Barahira, Maurizio Gallieni, Paloma Leticia Martin-Moreno, Gabriele Guglielmetti, Gabriella Guzzo, Nestor Toapanta, Maria Jose Soler, Antinus J. Luik, Willi H. M. van Kuijk, Lonneke W. H. Stikkelbroeck, Marc M. H. Hermans, Laurynas Rimsevicius, Marco Righetti, Mahmud Islam

**Affiliations:** 1 Department Internal Medicine, University Medical Center Groningen, University of Groningen, Hanzeplein 1, 9713 GZ Groningen, The Netherlands; 2 Department of Clinical Pharmacy & Pharmacology, University Medical Center Groningen, Groningen, The Netherlands; 3 Department of Nephrology, Radboud University Medical Center, Nijmegen, The Netherlands; 4 Medisch Centrum Leeuwarden, Leeuwarden, The Netherlands; 5 Grigore T Popa University of Medicine and Pharmacy, Iasi, Romania; 6 Dr Ci Parhon Hospital, Iasi, Romania; 7 Helsinki University Central Hospital and Helsinki University, Helsinki, Finland; 8 Zuyderland Ziekenhuis, Sittard-Geleen, The Netherlands; 9 Satakunta Central Hospital, Pori, Finland; 10 Tergooi Medical Center, Hilversum, The Netherlands; 11 ERA Registry, Amsterdam UMC Location University of Amsterdam, Medical Informatics, Meibergdreef 9, Amsterdam, The Netherlands; 12 Amsterdam Public Health Research Institute, Quality of Care, Amsterdam, The Netherlands; 13 Albert Schweitzer Hospital, Dordrecht, The Netherlands; 14 Ambroise Pare Hospital, APHP Paris-Saclay University, Boulogne Billancourt, France; 15 Amphia Hospital, Breda, The Netherlands; 16 Amsterdam UMC, Amsterdam, The Netherlands; 17 Antwerp University Hospital, Antwerp, Belgium; 18 Faculty of Medicine, Avicennes Military Hospital, Cadi Ayyad University, Marrakech, Morocco; 19 AZ Delta, Roeselare, Belgium; 20 B. Braun Avitum, Litomerice, Czech Republic; 21 Bellvitge University Hospital, Hospitalet de Llobregat, Barcelona, Spain; 22 Bernhoven Hospital, Uden, The Netherlands; 23 Bravis Hospital, Roosendaal/Bergen Op Zoom, The Netherlands; 24 Cantonal Hospital Zenica, Zenica, Bosnia and Herzegovina; 25 ‘Carol Davila’ University of Medicine and Pharmacy, Bucharest, Romania; 26 Emergency Clinical Hospital ‘Sf. Ioan’, Bucharest, Romania; 27 Catharina Hospital, Eindhoven, The Netherlands; 28 Central Clinical Hospital of the Ministry of Interior, Warsaw, Poland; 29 Centre Hospitalier du Nord, Ettelbruck, Luxembourg; 30 Centre of Postgraduate Medical Education, Warsaw, Poland; 31 Centrodial, São João da Madeira, Portugal; 32 Centro Hospitalar Vila Nova de Gaia/Espinho, Vila Nova de Gaia, Portugal; 33 City Hospital N.a. S.P. Botkin, Moscow, Russia; 34 City Hospital Zürich, Zurich, Switzerland; 35 Claude Galien Hospital Ramsay Santé, Quincy-Sous-Sénart, France; 36 Clínica de Hemodiálise de Felgueiras, Felgueiras, Portugal; 37 Clinical Centre of Vojvodina & Faculty of Medicine Novi Sad, University of Novi Sad, Novi Sad, Serbia; 38 Croatian Society of Nephrology, Dialysis and Transplantation, Zagreb, Croatia; 39 CWZ Nijmegen, Nijmegen, The Netherlands; 40 DaVita Geilenkirchen, Geilenkirchen, Germany; 41 DaVita, Wrocław, Poland; 42 1St Department of Internal Medicine, Medical School, Aristotle University of Thessaloniki, Thessaloniki, Greece; 43 Deventer Ziekenhuis, Deventer, The Netherlands; 44 Dianet Dialysis Center, Utrecht, The Netherlands; 45 Dialysis Center Bochum, Bochum, Germany; 46 Dnipro State Medical University, Dnipro, Ukraine; 47 Elyse Klinieken Voor Nierzorg, Kerkrade, The Netherlands; 48 Epidemiology Department, High Institute of Public Health-Alexandria University, Alexandria, Egypt; 49 Erasme Hospital, Brussels, Belgium; 50 Department of Internal Medicine, Erasmus MC Transplant Institute, University Medical Center Rotterdam, Rotterdam, The Netherlands; 51 Faculty of Medicine in Pilsen, Charles University, Pilsen, Czech Republic; 52 Faculty of Medicine-Alexandria University, Alexandria, Egypt; 53 Franciscus Gasthuis & Vlietland, Schiedam, The Netherlands; 54 Freeman Hospital, Newcastle Upon Tyne, UK; 55 Fundació Puigvert, Barcelona, Spain; 56 Fundación Jiménez Díaz, Madrid, Spain; 57 Gdansk Medical University, Gdansk, Poland; 58 Gelre Hospital, Apeldoorn, The Netherlands; 59 General Hospital of Athens “G. Gennimatas”, Athens, Greece; 60 General Hospital of Serres, Serres, Greece; 61 General University Hospital of Alexandroupolis, Alexandroupolis, Greece; 62 Grande Ospedale Metropolitano and CNR, Reggio Calabria, Italy; 63 Haaglanden Medisch Centrum, The Hague, The Netherlands; 64 Haga Hospital, The Hague, The Netherlands; 65 Hospital Clínic de Barcelona, Barcelona, Spain; 66 Hospital Curry Cabral-Central Lisbon University Hospital Center, Lisbon, Portugal; 67 Hospital das Clinicas, Universidade Federal de Minas Gerais, Belo Horizonte, Brazil; 68 Hospital del Mar, Barcelona, Spain; 69 Hospital de Santa Cruz, Centro Hospitalar de Lisboa Ocidental, Lisbon, Portugal; 70 Hospital Gelderse Vallei, Ede, The Netherlands; 71 Hospital General of Alicante, Alicante, Spain; 72 Hospital General Universitario Gregorio Marañón, Madrid, Spain; 73 Hospital Obispo Polanco, Salud Aragón, Spain; 74 Hospital Universitario Ramón y Cajal, Madrid, Spain; 75 Imelda Hospital, Bonheiden, Belgium; 76 Isala, Zwolle, The Netherlands; 77 Istanbul Faculty of Medicine, Istanbul University, Istanbul, Turkey; 78 Jeroen Bosch Ziekenhuis, Den Bosch, The Netherlands; 79 Klinikum Aschaffenburg-Alzenau, Aschaffenburg, Germany; 80 Leiden University Medical Center, Leiden, The Netherlands; 81 Lister Hospital, Stevenage, UK; 82 Lithuanian University of Health Sciences, Kaunas, Lithuania; 83 Luzerner Kantonsspital, Luzern, Switzerland; 84 Maasstad Ziekenhuis, Rotterdam, The Netherlands; 85 Maastricht University Medical Center, Maastricht, The Netherlands; 86 Manipal Hospital, Manipal, India; 87 Marche Nord Hospital, Pesaro, Italy; 88 Marmara University School of Medicine, Istanbul, Turkey; 89 Martini Ziekenhuis, Groningen, The Netherlands; 90 Maxima Medisch Centrum, Veldhoven, The Netherlands; 91 Meander Medisch Centrum, Amersfoort, The Netherlands; 92 Medical University Innsbruck, Innsbruck, Austria; 93 Medical University of Vienna, Vienna, Austria; 94 Moscow Regional Research and Clinical Institute, Moscow, Russia; 95 Necmettin Erbakan University Meram School of Medicine, Konya, Turkey; 96 Nephrology Unit, Department of Medicine and Surgery, University of Parma, Parma, Italy; 97 Nierenzentrum Reutlingen-Tübingen, Reutlingen, Germany; 98 Norwegian Renal Registry, Oslo University Hospital–Rikshospitalet, Olso, Norway; 99 Okinawa Chubu Hospital, Uruma, Japan; 100 OLVG, Amsterdam, The Netherlands; 101 Ospedale S. Maurizio Bolzano, Bolzano, Italy; 102 Padua University Hospital, Padua, Italy; 103 Radboud University Medical Center, Nijmegen, The Netherlands; 104 Regional Clinical Hospital, Yaroslavl, Russia; 105 Regional Hospital of Malaga, Malaga, Spain; 106 Rijnstate Hospital, Arnhem, The Netherlands; 107 RUDN University, Moscow, Russia; 108 Saint-Petersburg State University Hospital, Saint-Petersburg, Russia; 109 San Marco Hospital, University of Catania, Catania, Italy; 110 Saxenburgh Medisch Centrum, Hardenberg, The Netherlands; 111 Sint Antonius Ziekenhuis, Nieuwegein, The Netherlands; 112 Southern Health and Social Care Trust, Newry, Northern Ireland; 113 Spaarne Gasthuis, Haarlem, The Netherlands; 114 SPWSZ Hospital, Szczecinie, Poland; 115 St. Anna University Hospital, Ferrara, Italy; 116 St. Bassiano Hospital, Bassano del Grappo, Italy; 117 Streekziekenhuis Koningin Beatrix, Winterswijk, The Netherlands; 118 Swedish Renal Registry, Jönköping, Sweden; 119 Tehran University of Medical Sciences, Tehran, Iran; 120 Third Faculty of Medicine, Charles University, and Faculty Hospital Kralovske Vinohrady, Prague, Czech Republic; 121 Toledo University Hospital, Toledo, Spain; 122 Treant/Scheper Ziekenhuis, Emmen, The Netherlands; 123 Turgut Ozal Medical Center, Malatya, Turkey; 124 Université Catholique de Louvain, Cliniques Universitaires St Luc, Brussels, Belgium; 125 University Clinical Hospital of Santiago de Compostela, Santiago de Compostela, Spain; 126 University Clinical Hospital of Valladolid, Valladolid, Spain; 127 Universitary Hospital of Guadalajara, Guadalajara, Spain; 128 University Hospital Leuven, Leuven, Belgium; 129 University Hospital Brussels, Brussels, Belgium; 130 University Hospital Martin and Jessenius Faculty of Medicine Comenius University, Martin, Slovakia; 131 University Hospital Medical Center Verona, Verona, Italy; 132 University Hospitals of Coventry and Warwickshire NHS Trust, Coventry, UK; 133 University Hospital of Ioannina, Ioannina, Greece; 134 University Hospital of Patras, Patras, Greece; 135 University Medical Center Groningen, Groningen, The Netherlands; 136 University Medical Center Ljubljana, Ljubljana, Slovenia; 137 University Medical Centre Maribor, Maribor, Slovenia; 138 University Medical Center Utrecht, Utrecht, The Netherlands; 139 University of California Irvine School of Medicine, Orange, CA USA; 140 University of Genoa, Genoa, Italy; 141 University of Liège, Liège, Belgium; 142 University of Milan, Milan, Italy; 143 University of Navarra Clinic, Pamplona, Spain; 144 University of Piemonte Orientale, Novara, Italy; 145 Valais Hospital, Sion & Lausanne University Hospital, Lausanne, Switzerland; 146 Vall d’Hebron University Hospital, Barcelona, Spain; 147 VieCuri Medical Centre, Venlo, The Netherlands; 148 Vilnius University, Vilnius, Lithuania; 149 Vimercate Hospital, Vimercate, Italy; 150 Zonguldak Ataturk State Hospital, Zonguldak, Turkey

**Keywords:** Diseases, Infectious diseases, Kidney diseases

## Abstract

In the general population with COVID-19, the male sex is an established risk factor for mortality, in part due to a more robust immune response to COVID-19 in women. Because patients on kidney function replacement therapy (KFRT) have an impaired immune response, especially kidney transplant recipients due to their use of immunosuppressants, we examined whether the male sex is still a risk factor for mortality among patients on KFRT with COVID-19. From the European Renal Association COVID-19 Database (ERACODA), we examined patients on KFRT with COVID-19 who presented between February 1st, 2020, and April 30th, 2021. 1204 kidney transplant recipients (male 62.0%, mean age 56.4 years) and 3206 dialysis patients (male 61.8%, mean age 67.7 years) were examined. Three-month mortality in kidney transplant recipients was 16.9% in males and 18.6% in females (p = 0.31) and in dialysis patients 27.1% in males and 21.9% in females (p = 0.001). The adjusted HR for the risk of 3-month mortality in males (vs females) was 0.89 (95% CI 65, 1.23, p = 0.49) in kidney transplant recipients and 1.33 (95% CI 1.13, 1.56, p = 0.001) in dialysis patients (p_interaction_ = 0.02). In a fully adjusted model, the aHR for the risk of 3-month mortality in kidney transplant recipients (vs. dialysis patients) was 1.39 (95% CI 1.02, 1.89, p = 0.04) in males and 2.04 (95% CI 1.40, 2.97, p < 0.001) in females (p_interaction_ = 0.02). In patients on KFRT with COVID-19, the male sex is not a risk factor for mortality among kidney transplant recipients but remains a risk factor among dialysis patients. The use of immunosuppressants in kidney transplant recipients, among other factors, may have narrowed the difference in the immune response to COVID-19 between men and women, and therefore reduced the sex difference in COVID-19 mortality risk.

## Introduction

In the general population with COVID-19, men exhibit a higher risk of mortality compared with women. In a meta-analysis of over 3 million reported cases of COVID-19 globally, men were reported to have an almost 40% higher likelihood of mortality despite a similar likelihood of having confirmed COVID-19 when compared with women^1^. Previously it has been shown that women demonstrate a more robust immune response to COVID-19 compared with men and this is suggested to be one of the contributing factors to the observed sex differences in mortality risk^2^. This line of reasoning also suggests that in an immunocompromised patient population with COVID-19, the sex difference in mortality risk is narrowed or eliminated.

Patients on kidney function replacement therapy (KFRT) have an impaired immune response, especially kidney transplant recipients due to their use of immunosuppressants. Among kidney transplant recipients with COVID-19, some studies indeed showed no sex difference in mortality^3,4^. However, others demonstrated a higher risk of mortality in men compared with women^5^. Likewise, among dialysis patients with COVID-19, prior studies reported no to an almost twofold increased risk of mortality in men compared with women^6–9^. Importantly, these studies were in general relatively small in size and/or lacked information on key covariates, and therefore lacked power and careful control of these covariates when assessing sex-mortality relationships. Moreover, it remains unclear whether the sex difference in mortality risk differs between kidney transplant recipients and dialysis patients. A better understanding of potential sex-based differences in mortality risk among patients on KFRT with COVID-19 may guide more specific interventions and management of COVID-19 by incorporating sex considerations^10^.

Therefore, we examined whether sex is associated with the risk for mortality among patients on KFRT with COVID-19. For this study, we used data from the European Renal Association COVID-19 Database (ERACODA), the largest European database with detailed information on patient demographics, comorbidities, symptoms, laboratory results, and prospective follow-up for mortality in patients on KFRT with COVID-19.

## Materials and methods

### Study design and participants

ERACODA was established in March 2020 to study the prognosis and risk factors for mortality among kidney failure patients with COVID-19. Details of the database and the study design have been published previously^11^. Briefly, adult (≥ 18 years) patients either on dialysis (hemodialysis or peritoneal dialysis) or living with a functioning kidney allograft, who were diagnosed with COVID-19 based on a positive result on a real-time polymerase chain reaction assay or rapid antigen test of nasal and/or pharyngeal swab specimens, and/or compatible findings on CT scan or chest X-ray of the lungs were included. Data were voluntarily reported on outpatients and hospitalized patients by physicians responsible for their care. The database currently involves the cooperation of approximately 225 physicians representing over 140 centers in about 35 countries, mostly in Europe.

The database is hosted at the University Medical Center Groningen, The Netherlands. Data is recorded using REDCap software (Research Electronic Data Capture, Vanderbilt University Medical Center, Nashville, TN, USA) for data collection^12^. Patient information is stored pseudonymized. The study was approved by the Institutional Review Board (IRB) of the University Medical Center Groningen (Netherlands). Since the study did not involve identifiable private information and was observational in nature, a waiver of informed consent was granted by IRB of the University Medical Center Groningen in The Netherlands. Participating centers obtained study approval and waiver of consent from IRBs of their respective institute. All methods were performed per the relevant guidelines and regulations. The clinical and research activities being reported are consistent with the Principles of the Declaration of Istanbul as outlined in the 'Declaration of Istanbul on Organ Trafficking and Transplant Tourism'. No organs were procured from prisoners.

Detailed information was collected on patient characteristics (age, sex, race/ethnicity, height, weight, frailty, comorbidities, hospitalization, and medication use) and COVID-19-related characteristics (reason for COVID-19 screening, symptoms, vital signs, and laboratory test results) at presentation. Frailty was assessed using the Clinical Frailty Score developed by Rockwood et al.^13^. Obesity was defined as a body mass index ≥ 30 kg/m^2^. For the analysis, all patients who presented between February 1st, 2020, and April 30th, 2021, and for whom information on sex, the date of presentation, type of renal replacement therapy, and 3-month mortality was available were included (Fig. S1). The primary outcome was 3-month mortality. The secondary outcome was 28-day mortality.

### Statistical analysis

Baseline characteristics are presented by sex (male/female) for dialysis patients and kidney transplant recipients, separately. Continuous data are presented as mean (standard deviation (SD)) or as median (interquartile interval (IQI)) in case of a non-Gaussian distribution of data. Categorical data are presented as numbers (percentages). Baseline characteristics were compared between men and women using the independent sample t-test (in case of Gaussian distribution) or the Mann–Whitney U-test (in case of non-Gaussian distribution) for continuous variables and the Pearson Chi-2 test for categorical variables. The standardized difference in baseline characteristics between men and women for both continuous and categorical variables was also calculated. Standardized difference estimates are based only on sample statistics and are not directly influenced by sample size^14^. A standardized difference of 0.15 or more was used to indicate a relevant difference in baseline characteristics between men and women^15^.

To investigate the association between sex and mortality risk, hazard ratios (HRs) and 95% confidence intervals (CIs) were estimated for the association of sex (male versus female (reference)) with 3-month mortality using Cox proportional-hazards regression models. Multiple models were constructed to account for factors that may explain any observed difference in 3-month mortality between men and women. Model 1 was a crude (unadjusted) model. In Model 2 we adjusted for age (continuous) and clinical frailty score (Continuous). In Model 3, given sex-related differences in access to care^16–18^, we additionally adjusted for the reason for COVID-19 screening (symptoms-based screening/positive COVID-19 contact or routine screening). Model 4 was further adjusted for factors known to be associated with COVID-19 outcome, i.e. smoking (never, current, former), obesity (yes/no), hypertension (yes/no), diabetes (yes/no), heart failure (yes/no), chronic lung disease (yes/no), coronary artery disease (yes/no), and auto-immune disease (yes/no)^19^. In the final model (Model 5), we additionally adjusted for the duration of KFRT (years) and estimated the glomerular filtration rate. In dialysis patients, eGFR was assumed to be 0 for those with residual diuresis ≤ 200 mL/day and 5 mL/min/1.73 m^2^ for those with residual diuresis > 200 mL/day^20^. The proportional-hazards assumption was investigated by comparing a model with and without the interaction of log(time) with individual covariates. Cumulative incidence was plotted for 3-month mortality by sex. The Kolmogorov–Smirnov test was used to compare cumulative incidence between men and women.

To assess the robustness of the association between sex with mortality, we performed several additional analyses. First, to assess the consistency of our results across key subgroups we investigated results by subgroups of age (< 65/ ≥ 65 years), the reason for COVID-19 screening (symptoms-based screening/positive COVID-19 contact or routine screening), frailty (< 4/ ≥ 4), obesity (yes/no), hypertension (yes/no), and diabetes (yes/no). Second, assuming immunosuppressant use is among the main factors contributing to excess mortality in kidney transplant recipients compared with dialysis patients and immunosuppressant use affects men and women differently for the risk of mortality in presence of COVID-19, we investigated the association of type of KFRT with mortality by sex. Third, to further account for potential differences in access to care among men and women, we investigated the association between sex and 3-month mortality when starting follow-up from the date of symptom(s) onset. Fourth, to investigate potential sex differences in relatively short-term mortality risk, we examined the association of sex with 28-day mortality instead of 3-month mortality. Fifth, we investigated sex differences in mortality risk by hospitalization and intensive care unit admission status. Finally, to account for the potential influence of between-country differences on the sex-mortality relationship, we constructed a random intercept model with the country as a random factor in a multilevel mixed-effects parametric survival model.

All analyses were performed using Stata version 17.0 (College Station, TX). A 2-sided p-value less than 0.05 was adopted to indicate statistical significance.

## Results

### Baseline characteristics

Baseline characteristics of the study population by type of KFRT and sex are reported in Table 1. Among a total of 1204 kidney transplant recipients (mean age 56.4 years), 747 (62.0%) were men and 457 (38%) were women. Men on average had lower body mass index and clinical frailty scores compared with women, whereas the prevalence of prior smoking, hypertension, and coronary artery disease was higher among men. The prevalence of auto-immune diseases was higher among women compared with men. Presenting symptoms were largely comparable between men and women except that women more often reported nausea or vomiting.

**Table 1:** Baseline characteristics by sex in kidney transplant recipients and dialysis patients with COVID-19.

	Kidney transplant recipients (N = 1204)				Dialysis patients (N = 3206)			
	Women (N = 457)	Men (N = 747)	p-value	Std. difference	Women (N = 1225)	Men (N = 1981)	p-value	Std. difference
**Patient characteristics**								
Age, (years)	55.7 (14.1)	56.9 (13.7)	0.16	− 0.08	67.5 (14.6)	67.8 (14.2)	0.67	− 0.02
Body Mass Index, (kg/m^2^)	27.5 (5.8)	26.8 (4.3)	0.02	0.16	27.2 (6.5)	26.6 (4.8)	0.01	0.15
Caucasians, n (%)	354 (77.5)	616 (82.5)	0.07	0.10	978 (79.8)	1641 (82.8)	0.001	0.14
Tobacco use, n (%)			< 0.001	0.50			< 0.001	0.61
Current	12 (2.6)	35 (4.7)			53 (4.3)	154 (7.8)		
Prior	53 (11.6)	206 (27.6)			111 (9.1)	501 (25.3)		
Never	287 (62.8)	311 (41.6)			618 (50.4)	510 (25.7)		
Unknown	105 (23.0)	195 (26.1)			443 (36.2)	816 (41.2)		
Reason for screening^a^, n(%)			0.48	0.10			0.41	0.07
Symptoms only	332 (72.6)	533 (71.3)			580 (47.3)	978 (49.4)		
Symptoms and COVID + contact	49 (10.7)	85 (11.4)			146 (11.9)	212 (10.7)		
COVID + contact only	19 (4.2)	29 (3.9)			113 (9.2)	160 (8.1)		
Routine	16 (3.5)	15 (2.0)			104 (8.5)	469 (8.2)		
Clinical frailty scale, AU	3.2 (1.6)	2.8 (1.4)	< 0.001	0.28	4.2 (1.8)	3.9 (1.8)	< 0.001	0.15
Comorbidities, n (%)								
Hypertension	352 (77.0)	631 (84.5)	0.001	− 0.19	969 (79.2)	1607 (81.1)	0.18	− 0.05
Diabetes mellitus	130 (28.6)	249 (33.4)	0.08	− 0.10	494 (40.4)	871 (44.0)	0.04	− 0.07
Coronary artery disease	49 (10.8)	162 (21.7)	< 0.001	− 0.30	354 (28.9)	742 (37.6)	< 0.001	− 0.18
Heart failure	34 (7.5)	68 (9.1)	0.32	− 0.06	281 (23.0)	464 (23.5)	0.73	− 0.01
Chronic lung disease	33 (7.3)	54 (7.2)	0.99	0.00	136 (11.1)	274 (13.9)	0.02	− 0.08
Active malignancy	18 (4.0)	29 (3.9)	0.96	0.00	50 (4.1)	135 (6.8)	0.001	− 0.12
Auto-immune disease	34 (7.5)	27 (3.9)	0.01	0.15	62 (5.1)	60 (3.0)	0.004	0.10
Primary kidney disease, n (%)								
Primary glomerulonephritis	66 (14.5)	140 (19.1)	0.04	− 0.12	120 (10.2)	237 (12.6)	0.04	− 0.08
Pyelonephritis	15 (3.3)	13 (1.8)	0.09	0.10	23 (1.9)	25 (1.3)	0.18	0.05
Interstitial nephritis	20 (4.4)	19 (2.6)	0.09	0.10	35 (3.0)	51 (2.7)	0.69	0.01
Hereditary kidney disease	56 (12.3)	99 (13.5)	0.56	− 0.03	88 (7.5)	113 (6.0)	0.12	0.06
Congenital diseases	24 (5.3)	29 (4.0)	0.28	0.06	22 (1.9)	24 (1.3)	0.20	0.05
Vascular diseases	38 (8.4)	52 (7.1)	0.42	0.05	220 (18.6)	346 (18.4)	0.89	0.01
Sec. glomerular disease	30 (6.6)	42 (5.7)	0.54	0.04	107 (9.1)	147 (7.8)	0.23	0.04
Diabetic kidney disease	41 (9.0)	86 (11.7)	0.14	− 0.09	260 (22.0)	421 (22.4)	0.79	− 0.01
Other	98 (21.6)	168 (22.9)	0.59	− 0.03	213 (18.0)	374 (19.9)	0.20	− 0.05
Unknown	66 (14.5)	85 (11.6)	0.14	0.09	93 (7.9)	140 (7.5)	0.67	0.02
Dialysis duration, years	–	–	–	–	3 (1, 7)	3 (1, 5)	0.10	0.13
Transplant duration, n (%)			0.49	0.07				
< 1 year	33 (7.2)	49 (6.6)			–	–	–	–
1–5 years	170 (37.2)	304 (40.7)			–	–	–	–
> 5 years	250 (54.7)	389 (52.1)			–	–	–	–
**Medications**^**b**^**, n (%)**								
Immunosuppressants			0.58	− 0.01				
Monotherapy	14 (3.1)	19 (2.6)			–	–	–	–
Dual therapy	152 (33.6)	232 (31.4)			–	–	–	–
Triple therapy	286 (63.3)	489 (66.1)			–	–	–	–
RAAS inhibitors	142 (35.9)	281 (44.5)	0.01	− 0.18	209 (23.4)	439 (30.8)	< 0.001	− 0.17
**Disease characteristics**								
Presenting symptoms, n (%)								
Sore throat	82 (19.2)	112 (16.5)	0.26	0.07	140 (14.6)	192 (13.0)	0.25	0.05
Cough	265 (60.2)	438 (61.5)	0.66	0.03	471 (47.0)	765 (48.9)	0.36	0.04
Shortness of breath	168 (38.1)	297 (41.9)	0.20	0.08	318 (31.6)	502 (32.0)	0.81	0.01
Fever	281 (63.3)	497 (69.5)	0.03	0.13	506 (50.3)	873 (55.6)	0.01	0.16
Headache	106 (25.1)	140 (21.0)	0.12	0.10	131 (13.7)	122 (8.3)	< 0.001	0.18
Nausea or vomiting	89 (20.7)	84 (12.3)	< 0.001	0.23	133 (13.5)	151 (9.8)	0.004	0.12
Diarrhoea	124 (28.7)	177 (25.8)	0.28	0.07	132 (13.3)	196 (12.8)	0.68	0.02
Myalgia or arthralgia	135 (31.8)	208 (31.3)	0.88	0.01	214 (22.3)	322 (21.7)	0.74	0.01
Vital signs								
Temperature, °C	37.6 (1.1)	37.5 (1.1)	0.30	0.07	37.3 (1.0)	37.5 (1.0)	0.001	− 0.15
Respiration rate, /min	20.5 (6.6)	21.0 (7.3)	0.30	− 0.07	18.4 (4.8)	18.6 (5.1)	0.35	− 0.04
O_2_ saturation room air, %	94.3 (6.4)	93.9 (6.6)	0.36	0.06	94.1 (5.1)	93.7 (5.8)	0.16	0.06
Systolic BP, mm Hg	132.0(21.9)	133.5 (21.0)	0.37	− 0.07	137.0 (25.0)	136.3 (25.9)	0.54	0.03
Diastolic BP, mm Hg	77.8 (14.2)	78.4 (13.9)	0.60	− 0.04	73.4 (15.0)	73.6 (15.5)	0.85	− 0.01
Pulse rate, BPM	89.0 (16.7)	86.3 (16.9)	0.04	0.16	80.2 (14.2)	81.8 (16.0)	0.03	− 0.11
Laboratory test results								
eGFR, ml/min/1.73m^2^	41.7 (23.8)	42.9 (23.7)	0.59	− 0.05	–	–	–	–
Lymphocytes, ×1000/µL	0.8 (0.5, 1.3)	0.8 (0.5, 1.3)	0.60	− 0.06	0.9 (0.6, 1.3)	0.9 (0.6, 1.3)	0.46	0.01
CRP, mg/L	72 (25, 147)	83 (33, 187)	0.05	− 0.14	68 (20, 190)	82 (27, 240)	0.03	− 0.15

Continuous variables are reported as mean (standard deviation) or median Interquartile interval). Groups were compared using independent sample t-test, Mann–Whitney U-test, or Pearson Chi-square test as appropriate. Obesity is defined as BMI > 30 kg/m^2^.

Std., standardized; °C, degree Celsius; O_2_, oxygen; BP, blood pressure; BPM, beats per minute; eGFR, estimated glomerular filtration rate; CRP, C-reactive protein; RAAS = Renin–angiotensin–aldosterone system.

^a^Total number may not add up due to missingness (126 missing in kidney transplant recipients, 751 missing in dialysis patients).

^b^ In those hospitalized.

Among 3206 dialysis patients (mean age 67.7 years), 1981 (61.8%) were men and 1225 (38.2%) were women. Similar to kidney transplant recipients, the prevalence of prior smoking and chronic artery disease was higher among men compared with women and again the prevalence of auto-immune diseases was higher among women. Men more often had fever at presentation and the level of C-reactive protein was also higher among men compared with women.

### Three-month mortality

**Figure 1:**
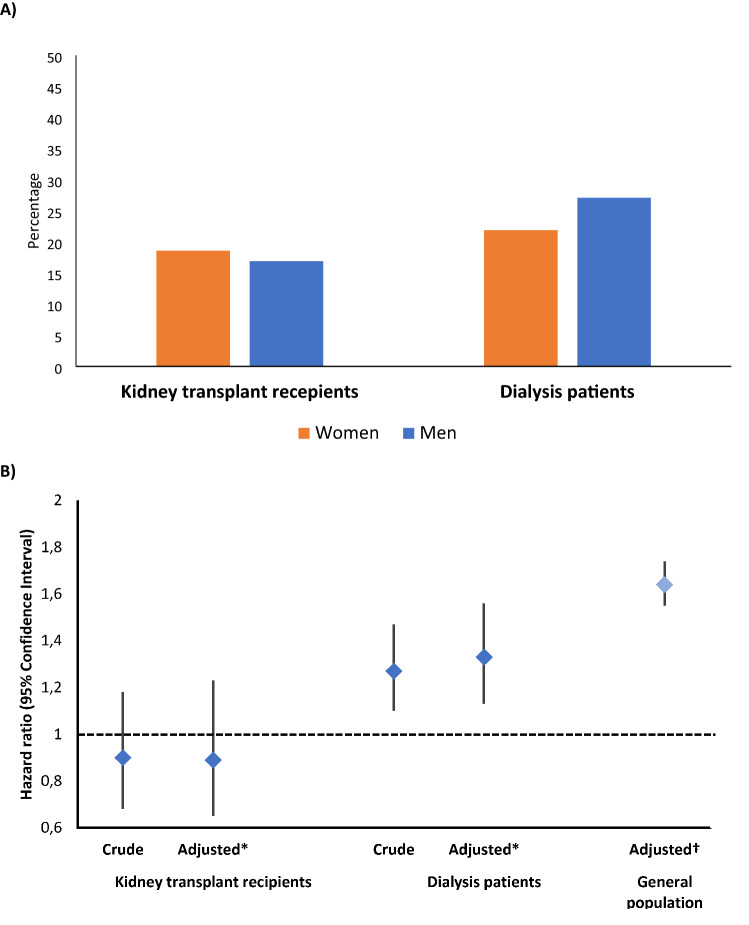
Percentage mortality by sex and type of kidney function replacement therapy (**A**) and hazard ratio for the association of sex (male vs. female (reference)) with 3-month mortality by type of kidney function replacement therapy (**B**). *Adjusted for: age (continuous), clinical frailty score (continuous), the reason for COVID-19 screening (symptoms-based screening, positive COVID-19 contact or routine screening), smoking (never, current, former), obesity (yes/no), hypertension (yes/no), diabetes (yes/no), heart failure (yes/no), chronic lung disease (yes/no), coronary artery disease (yes/no), and auto-immune disease (yes/no), duration of kidney function replacement therapy (years) and estimated glomerular filtration rate (continuous). ^†^Adjusted estimate from literature (Nat Commun 2020: https://pubmed.ncbi.nlm.nih.gov/33298944/).

In kidney transplant recipients, 16.9% of men and 18.6% of women died within 3 months of presentation (p = 0.31). Cumulative mortality incidence was similar between men and women (p = 0.57) (Fig. S2). In a crude model, the HR for the risk of 3-month mortality in men (vs women) was 0.90 (95% CI 0.68, 1.18; p = 0.43). In the final multivariable model adjusted model (Model 5), the HR for the risk of 3-month mortality in men vs women was 0.89 (95% CI 0.65, 1.23; p = 0.49) (Table 2; Fig. 1).

**Table 2:** Association of sex with 3-month mortality in kidney transplant recipients and dialysis patients with COVID-19 (presented are hazard ratios with 95% confidence intervals).

Transplant recipients (N = 1204)	Women (N = 457)	Men (N = 747)	p-value
Event, n (%)	85 (18.6)	126 (16.9)	
Model 1	Ref.	0.90 (0.68, 1.18)	0.43
Model 2	Ref.	0.97 (0.73, 1.29)	0.86
Model 3	Ref.	0.96 (0.73, 1.28)	0.80
Model 4	Ref.	0.87 (0.63, 1.19)	0.37
Model 5	Ref.	0.89 (0.65, 1.23)	0.49

Dialysis patients (N = 3206)	Women (N = 1225)	Men (N = 1,981)	p-value
Event, n (%)	268 (21.9)	536 (27.1)	
Model 1	Ref.	1.27 (1.10, 1.47)	0.001
Model 2	Ref.	1.41 (1.21, 1.64)	< 0.001
Model 3	Ref.	1.40 (1.20, 1.63)	< 0.001
Model 4	Ref.	1.32 (1.13, 1.56)	0.001
Model 5	Ref.	1.33 (1.13, 1.56)	0.001

Model 1: crude.

Model 2: Model 1 + age (continuous), clinical frailty score (continuous).

Model 3: Model 2 + the reason for COVID-19 screening (symptoms-based screening, positive COVID-19 contact or routine screening).

Model 4: Model 3 + smoking (never, current, former), obesity (yes/no), hypertension (yes/no), diabetes (yes/no), heart failure (yes/no), chronic lung disease (yes/no), coronary artery disease (yes/no), and auto-immune disease (yes/no).

Model 5: Model 4 + duration of kidney function replacement therapy (years) and estimated glomerular filtration rate (continuous).

(p-for interaction between sex and type of kidney function replacement therapy = 0.02 in fully adjusted model for 3 month mortality).

In dialysis patients, 27.1% of men and 21.9% of women died within 3 months of presentation (p = 0.001). Cumulative mortality incidence was higher in men compared with women (p < 0.001) (Fig. S2). In a crude model, the HR for the risk of 3-month mortality in men vs women was 1.27 (95% CI 1.10, 1.47; p = 0.001) and in the fully adjusted model, it was 1.33 (95% CI 1.13, 1.56, p = 0.001) in dialysis patients (Table 2; Fig. 1).

The interaction between sex and type of KFRT for mortality risk was statistically significant (p for interaction = 0.02). No violation of the proportional hazards assumption was noted in the fully adjusted model for kidney transplant recipients nor for dialysis patients (p-value for difference between the model with and without interaction between log(time) and covariates being 0.41 in case of kidney transplant recipients and 0.75 in case of dialysis patients).

### Additional analyses

**Figure 2:**
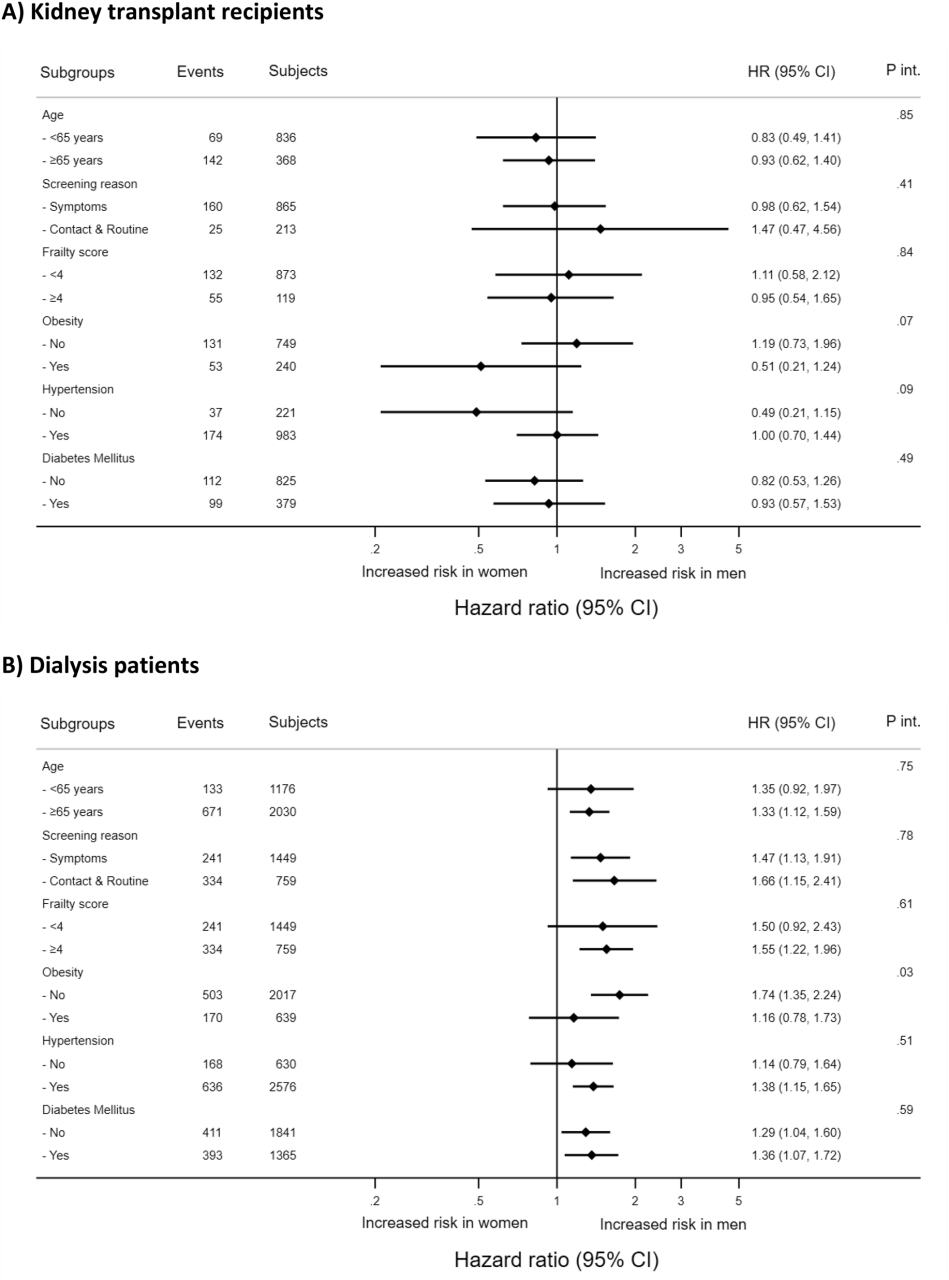
Association of sex with 3-month mortality in kidney transplant recipients (**A**) and dialysis patients (**B**) with COVID-19 by key subgroups (presented are hazard ratios for male versus female with 95% confidence intervals, and p-values for interaction).

The observed association between sex and 3-month mortality risk in kidney transplant recipients and dialysis patients was consistent across all examined subgroups except across the subgroup of obesity (no/yes) in dialysis patients where the association was particularly evident among non-obese patients (Fig. 2).

In a fully adjusted model, the HR for the risk of 3-month mortality in kidney transplant recipients (vs. dialysis patients) was 1.39 (95% CI 1.02, 1.89, p = 0.04) among men and was 2.04 (95% CI 1.40, 2.97, p < 0.001) among women (p for interaction = 0.02) (Table 3). When the follow-up period was considered to start from the date of symptom(s) onset, the association between sex and 3-month mortality remained statistically non-significant among kidney transplant recipients and statistically significant among dialysis patients (Table S1). When investigating the association between sex and mortality for 28-day mortality, by hospitalization status or by ICU admission status, results were essentially similar to our main findings (Tables S2, S3, and S4 respectively). Finally, the observed association between sex and 3-month mortality risk in kidney transplant recipients and dialysis patients remained unchanged when accounting for potential between-country differences in the relationship between sex and mortality (Table S5).

**Table 3:** Association of type of renal replacement therapy (kidney transplant vs dialysis) with 3-month mortality in men (upper panel) and women (lower panel) with COVID-19 (presented are hazard ratios with 95% confidence intervals).

Men (N = 2728)	Dialysis patients (N = 1981)	Kidney transplant recipients (N = 747)	p-value
Event, n (%)	536 (27.1)	126 (16.9)	
Model 1	Ref.	0.57 (0.47, 0.69)	< 0.001
Model 2	Ref.	1.25 (1.01, 1.55)	0.04
Model 3	Ref.	1.19 (0.96, 1.47)	0.13
Model 4	Ref.	1.20 (0.97, 1.50)	0.09
Model 5	Ref.	1.39 (1.02, 1.89)	0.04

Women (N = 1682)	Dialysis patients (N = 1225)	Kidney transplant recipients (N = 457)	p-value
Event, n (%)	268 (21.9)	85 (18.6)	
Model 1	Ref.	0.81 (0.63, 1.03)	0.09
Model 2	Ref.	1.63 (1.25, 2.13)	< 0.001
Model 3	Ref.	1.54 (1.17, 2.01)	0.002
Model 4	Ref.	1.64 (1.24, 2.17)	0.001
Model 5	Ref.	2.04 (1.40, 2.97)	< 0.001

Model 1: crude.

Model 2: Model 1 + age (continuous), clinical frailty score (continuous).

Model 3: Model 2 + the reason for COVID-19 screening (symptoms-based screening/positive COVID-19 contact or routine screening).

Model 4: Model 3 + smoking (never, current, former), obesity (yes/no), hypertension (yes/no), diabetes (yes/no), heart failure (yes/no), chronic lung disease (yes/no), coronary artery disease (yes/no), and auto-immune disease (yes/no).

Model 5: Model 4 + duration of kidney function replacement therapy (years) and estimated glomerular filtration rate (continuous).

(p-for interaction between sex and type of kidney function replacement therapy = 0.02 in fully adjusted model for 3 month mortality).

## Discussion

In this large study of patients on KFRT with COVID-19, men were at higher risk of mortality in dialysis patients, whereas mortality risk was similar in males and females in kidney transplant recipients. The observed association between sex and mortality risk in dialysis and transplant patients was consistent across key subgroups except across subgroups according to body mass index in dialysis patients where the increased risk of mortality in men was particularly high among non-obese patients. Importantly, when men and women were investigated separately for the association of type of KFRT with mortality, there was less difference in the adjusted risk of mortality between kidney transplant recipients and dialysis patients among men compared with women.

Studies in the early phase of the pandemic that investigated the sex-mortality relationship among kidney transplant recipients were relatively small and reported no difference in mortality between men and women. However, it remained unclear whether men and women continued to exhibit a similar risk of mortality as more data accumulated. A recent large retrospective study among solid organ transplant recipients suggested a 47% higher risk of mortality in men compared with women in kidney transplant recipients when accounting for differences in patient characteristics^5^. Unfortunately, due to the retrospective nature, this study had a considerable number of missing records for comorbidities and due to miscoding, a substantial number of these patients may have been misclassified as not having comorbidity. It is worth noting that in the case of other infectious diseases, such as influenza, where men are reported to have an increased risk of mortality in the general population^21,22^, sex has also been reported not to be associated with mortality in immunocompromised study populations^23^.

The use of immunosuppressants may be one of the reasons for the lack of difference in risk of mortality between men and women in kidney transplant recipients with COVID-19. Previously it has been demonstrated that in the general population with COVID-19, men have increased plasma levels of innate immune cytokines such as IL-8 and IL-18 along with more robust induction of monocytes, whereas women show more robust T cell activation compared with men^2^. This study also demonstrated that higher levels of innate immune cytokines and poor T-cell response were associated with poor outcomes, suggesting a more robust immune response to COVID-19 potentially contributes to the survival advantage among women^2^. However, among kidney transplant recipients, which typically are on maintenance immunosuppression, any survival advantage due to a robust immune response may be mitigated.

Among dialysis patients with COVID-19, our results are in line with other larger studies published later in the COVID-19 pandemic which also showed an increased risk of mortality in men compared with women^8,9^. Earlier studies, however, did not specifically aim to investigate the sex-mortality relationship and lacked careful control of factors that may explain the excess risk of mortality among men^6–9,24^. Consequently, it was unclear whether the association between sex and mortality in dialysis patients was independent of potential sex differences in comorbidities and high-risk behaviours including those related to access to health care. Our study demonstrated that the association between sex and mortality persists independent of comorbidities and factors related to healthcare access, and in our study, we additionally accounted for clinical frailty score and potential sex differences in the time from symptom(s) onset to clinical presentation to limit the possibility of residual influence from comorbidities and factors related to health care access.

Dialysis patients have impaired immune function which may influence the sex-mortality relationship in this population compared with the general population when infected with COVID-19. Among dialysis patients in our study, the risk of mortality was about 30% higher in men compared to women. In the general population with COVID-19, a meta-analysis including 92 studies and 3,111,714 subjects reported an almost 40% higher likelihood of mortality in men^1^. When this analysis was repeated after accounting for reporting bias, the likelihood of mortality in men was estimated to be even about 64% higher in men compared with women. Other studies in the general population with COVID-19 that were not included in the aforementioned meta-analysis, with similar mean age and design as our study, reported an almost twofold higher adjusted risk of mortality in men compared with women^25^. These mortality risks appear higher than the 1.39 increased mortality risk that we found in male versus female patients on dialysis. These data suggest that the sex difference in mortality risk among dialysis patients may be narrower compared to the sex difference in mortality risk among the general population with COVID-19.

Our results also demonstrated that the absolute risk of mortality is lower in kidney transplant recipients compared with dialysis patients. However, it should be noted that after adjustment for differences in age, frailty, and comorbidities between these two patient groups, the risk of mortality is actually higher among kidney transplant recipients compared with dialysis patients^20^. Differences in risk of mortality by type of KFRT and sex were apparent when we analyzed male–female kidney transplant recipients and dialysis patients together in one combined dataset (Table S6).

Our findings imply that sex may be an important factor in the management of patients on KFRT with COVID-19. Male dialysis patients should be informed about their higher risk of complications compared with females when infected with COVID-19 and be advised when in doubt to seek medical attention in the case of (suspected) COVID-19.

The present study has a number of strengths. This study includes detailed information on key patient and disease characteristics and prospective information on mortality from a large number of dialysis patients and kidney transplant recipients with COVID-19, which allowed a comprehensive assessment of the sex-mortality association including a direct comparison of the sex-mortality relationship between kidney transplant recipients and dialysis patients. This study was also able to investigate the association between sex and mortality by reason for COVID-19 screening which is particularly relevant given the sex difference in health care seeking behaviour^16–18^. However, this study also has limitations. First, we did not collect the information on viral load. Therefore, we were not able to investigate whether males and females differed in their viral load. Second, we only had data available from patients infected with wild-type or early variants of COVID-19 (e.g. alpha and delta)^26^. To our knowledge, there has also been no evidence that one viral strain/mutation affected the sex difference in mortality risk more than others. Moreover, data in our study were collected before mass vaccination was rolled out and before the efficacy of any of the currently known pharmacological treatments (e.g. steroid, remdesivir, and/or tocilizumab) was established. This allowed us to investigate the sex-mortality association in a homogeneous population that is unlikely to be influenced by any possible sex difference in vaccination rate or response, or in medication use or efficacy. Third, given the observational nature of the study design it was not possible to reliably investigate whether the dose and/or type of immunosuppressant influenced the sex-mortality relationship among kidney transplant recipients. Fourth, because reporting was voluntary, the included patients may not be completely representative of the overall population of KFRT patients with COVID-19. However, it should be noted that COVID-19 case-fatality rates and relative risk of mortality among men (vs. women) among dialysis patients in our study are comparable to those reported in KFRT registry studies that include non-selected populations, but lack detailed information for adjustment as we have in our study^9^.

In conclusion, among patients on KFRT with COVID-19, the male sex is not a risk factor for mortality in kidney transplant recipients but remains a risk factor in dialysis patients. The use of immunosuppressants in kidney transplant recipients, among other factors, may have narrowed the difference in the immune response to COVID-19 between men and women, and therefore reduced the sex difference in the risk of COVID-19 mortality.

## Supplementary Information

Supplementary Information.

## Data Availability

The dataset analysed during the current study is available from the corresponding author on reasonable request.
