# Supplementary material for: Sex differences in COVID-19 mortality risk in patients on kidney function replacement therapy
Source: Sci Rep. 2022 Oct 26;12:17978. doi: 10.1038/s41598-022-22657-4 (PMC9606116; doi:10.1038/s41598-022-22657-4)

**SUPPLEMENTARY MATERIAL**

**Sex differences in COVID-19 mortality risk in patients on**

**kidney function replacement therapy**

**Contents:**

**Table S1:** Association of sex with 3-month mortality in kidney transplant recipients and dialysis patients with COVID-19 when starting follow-up from date of symptom(s) onset (presented are hazard ratios with 95% confidence intervals)

**Table S2:** Association of sex with 28-day mortality in dialysis patients and kidney transplant recipients with COVID-19 (presented are hazard ratios with 95% confidence intervals)

**Table S3:** Association of sex with mortality in dialysis patients and kidney transplant recipients with COVID-19 by hospitalization status (presented are hazard ratios with 95% confidence intervals)

**Table S4:** Association of sex with mortality in dialysis patients and kidney transplant recipients with COVID-19 by intensive care unit admission status among hospitalized patients (presented are hazard ratios with 95% confidence intervals)

**Table S5:** Association of sex with 3-month mortality in kidney transplant recipients and dialysis patients with COVID-19 when accounting for country effect (presented are hazard ratios with 95% confidence intervals)

**Table S6:** Association of sex and type of kidney function replacement therapy with mortality among patients on kidney function replacement therapy with COVID-19

**Figure S1:** Flow chart of the number of ERACODA participants included in the current analyses

**Figure S2:** Cumulative incidence of three-month mortality by sex for kidney transplant recipients (Panel A) and Dialysis patients (Panel B)

**Table S1: Association of sex with 3-month mortality in kidney transplant recipients and dialysis patients with COVID-19 when starting follow-up from date of symptom(s) onset (presented are hazard ratios with 95% confidence intervals)**

| **Transplant recipients (N=1,204)** |  | **Women (N=457)** | **Men (N=747)** | **p-value** |
| --- | --- | --- | --- | --- |
| **Event, n (%)** |  | **85 (18.6)** | **126 (16.9)** |  |
| Model 1 |  | Ref. | 0.83 (0.62, 1.10) | 0.19 |
| Model 2 |  | Ref. | 0.91 (0.67, 1.22) | 0.52 |
| Model 3 |  | Ref. | 0.92 (0.68, 1.24) | 0.57 |
| Model 4 |  | Ref. | 0.85 (0.60, 1.19) | 0.33 |
| Model 5 |  | Ref. | 0.86 (0.61, 1.21) | 0.39 |
| **Dialysis patients (N=3,206)** |  | **Women (N=1,225)** | **Men (N=1,981)** | **p-value** |
| **Event, n (%)** |  | **267 (21.8)** | **536 (27.1)** |  |
| Model 1 |  | Ref. | 1.33 (1.12, 1.57) | 0.001 |
| Model 2 |  | Ref. | 1.52 (1.28, 1.80) | <0.001 |
| Model 3 |  | Ref. | 1.51 (1.27, 1.79) | <0.001 |
| Model 4 |  | Ref. | 1.42 (1.18, 1.70) | <0.001 |
| Model 5 |  | Ref. | 1.42 (1.18, 1.70) | <0.001 |

Model 1: crude

Model 2: Model 1 + age (continuous), clinical frailty score (continuous)

Model 3: Model 2 + the reason for COVID-19 screening (symptoms-based screening/positive COVID-19 contact or routine screening)

Model 4: Model 3 + smoking (never, current, former), obesity (yes/no), hypertension (yes/no), diabetes (yes/no), heart failure (yes/no), chronic lung disease (yes/no), coronary artery disease (yes/no), and auto-immune disease (yes/no)

Model 5: Model 4 + duration of kidney function replacement therapy (years) and estimated glomerular filtration rate (continuous)

(p-for interaction between sex and type of kidney function replacement therapy=0.004 in fully adjusted model for three month mortality)

**Table S2: Association of sex with 28-day mortality in dialysis patients and kidney transplant recipients with COVID-19 (presented are hazard ratios with 95% confidence intervals)**

| **Transplant recipients**  **(N=1,204)** |  | **Women**  **(N=457)** | **Men**  **(N=747)** | **p-value** |
| --- | --- | --- | --- | --- |
| **Death, n (%)** |  | **73 (16.0)** | **104 (13.9)** |  |
| Model 1 |  | Ref. | 0.86 (0.64, 1.16) | 0.22 |
| Model 2 |  | Ref. | 0.92 (0.68, 1.26) | 0.62 |
| Model 3 |  | Ref. | 0.91 (0.67, 1.25) | 0.57 |
| Model 4 |  | Ref. | 0.80 (0.56, 1.13) | 0.20 |
| Model 5 |  | Ref. | 0.82 (0.58, 1.17) | 0.27 |
| **Dialysis patients**  **(N=3,206)** |  | **Women**  **(N=1,225)** | **Men**  **(N=1,981)** | **p-value** |
| **Death, n (%)** |  | **235 (19.2)** | **451 (22.8)** |  |
| Model 1 |  | Ref. | 1.21 (1.03, 1.41) | 0.02 |
| Model 2 |  | Ref. | 1.31 (1.12, 1.55) | 0.001 |
| Model 3 |  | Ref. | 1.30 (1.10, 1.53) | 0.002 |
| Model 4 |  | Ref. | 1.23 (1.03, 1.46) | 0.02 |
| Model 5 |  | Ref. | 1.23 (1.04, 1.47) | 0.02 |

Model 1: crude

Model 2: Model 1 + age (continuous), clinical frailty score (continuous)

Model 3: Model 2 + the reason for COVID-19 screening (symptoms-based screening/positive COVID-19 contact or routine screening)

Model 4: Model 3 + smoking (never, current, former), obesity (yes/no), hypertension (yes/no), diabetes (yes/no), heart failure (yes/no), chronic lung disease (yes/no), coronary artery disease (yes/no), and auto-immune disease (yes/no)

Model 5: Model 4 + duration of kidney function replacement therapy (years) and estimated glomerular filtration rate (continuous)

(p-for interaction between sex and type of kidney function replacement therapy=0.04 in fully adjusted model for 28-day mortality)

**Table S3: Association of sex with mortality in dialysis patients and kidney transplant recipients with COVID-19 by hospitalization status (presented are hazard ratios with 95% confidence intervals)**

| **Kidney Transplant recipients** | | | | |
| --- | --- | --- | --- | --- |
| **Hospitalized (N=836)** |  | **Women (N=319)** | **Men (N=517)** | **p-value** |
| **Death, n (%)** |  | **80 (25.1)** | **122 (23.6)** |  |
| Model 1 |  | Ref. | 0.93 (0.70, 1.24) | 0.63 |
| Model 2 |  | Ref. | 0.97 (0.72, 1.30) | 0.83 |
| Model 3 |  | Ref. | 0.96 (0.71, 1.28) | 0.76 |
| Model 4 |  | Ref. | 0.89 (0.64, 1.24) | 0.48 |
| Model 5 |  | Ref. | 0.92 (0.66, 1.29) | 0.63 |
| **Non-hospitalized (N=367)** |  | **Women (N=137)** | **Men (N=230)** | **p-value** |
| **Death, n (%)** |  | **5 (3.6)** | **4 (1.7)** |  |
| Model 1 |  | Ref. | 0.46 (0.12, 1.73) | 0.25 |
| Model 2 |  | Ref. | 0.71 (0.15, 3.28) | 0.66 |
| Model 3 |  | Ref. | NR |  |
| Model 4 |  | Ref. | NR |  |
| Model 5 |  | Ref. | NR |  |
| **Dialysis patients** | | | | |
| **Hospitalized (N=1,797)** |  | **Women (N=660)** | **Men (N=1,137)** | **p-value** |
| **Death, n (%)** |  | **215 (32.6)** | **418 (36.8)** |  |
| Model 1 |  | Ref. | 1.15 (0.97, 1.35) | 0.10 |
| Model 2 |  | Ref. | 1.24 (1.05, 1.46) | 0.01 |
| Model 3 |  | Ref. | 1.24 (1.05, 1.46) | 0.01 |
| Model 4 |  | Ref. | 1.19 (1.00, 1.39) | 0.05 |
| Model 5 |  | Ref. | 1.16 (0.97, 1.39) | 0.09 |
| **Non-hospitalized (N=1,405)** |  | **Women (N=563)** | **Men (N=842)** | **p-value** |
| **Death, n (%)** |  | **53 (9.4)** | **118 (14.0)** |  |
| Model 1 |  | Ref. | 1.53 (1.11, 2.12) | 0.01 |
| Model 2 |  | Ref. | 1.75 (1.15, 2.44) | 0.001 |
| Model 3 |  | Ref. | 1.72 (1.24, 2.40) | 0.001 |
| Model 4 |  | Ref. | 1.58 (1.11, 2.25) | 0.01 |
| Model 5 |  | Ref. | 1.58 (1.11, 2.26) | 0.01 |

NR: Not reliable (due to fewer events)

Model 1: crude

Model 2: Model 1 + age (continuous), clinical frailty score (continuous)

Model 3: Model 2 + the reason for COVID-19 screening (symptoms-based screening/positive COVID-19 contact or routine screening)

Model 4: Model 3 + smoking (never, current, former), obesity (yes/no), hypertension (yes/no), diabetes (yes/no), heart failure (yes/no), chronic lung disease (yes/no), coronary artery disease (yes/no), and auto-immune disease (yes/no)

Model 5: Model 4 + duration of kidney function replacement therapy (years) and estimated glomerular filtration rate (continuous)

(p-for interaction between sex and hospitalization in kidney transplant recipients=0.43 and in dialysis patients=0.10)

(hospitalization information missing for 1 kidney transplant recipient and 4 dialysis patients)

**Table S4: Association of sex with mortality in dialysis patients and kidney transplant recipients with COVID-19 by intensive care admission status among hospitalized patients (presented are hazard ratios with 95% confidence intervals)**

| **Kidney Transplant recipients (N=834)** | | | | |
| --- | --- | --- | --- | --- |
| **ICU admitted (N=187)** |  | **Women (N=71)** | **Men (N=116)** | **p-value** |
| **Death, n (%)** |  | **38 (53.5)** | **58 (50.0)** |  |
| Model 1 |  | Ref. | 0.83 (0.55, 1.25) | 0.37 |
| Model 2 |  | Ref. | 0.77 (0.51, 1.16) | 0.21 |
| Model 3 |  | Ref. | 0.77 (0.51, 1.17) | 0.23 |
| Model 4 |  | Ref. | 0.59 (0.36, 0.97) | 0.04 |
| Model 5 |  | Ref. | 0.62 (0.37, 1.02) | 0.06 |
| **Non-ICU admitted (N=647)** |  | **Women (N=246)** | **Men (N=401)** | **p-value** |
| **Death, n (%)** |  | **41 (16.7)** | **64 (16.0)** |  |
| Model 1 |  | Ref. | 0.97 (0.66, 1.44) | 0.88 |
| Model 2 |  | Ref. | 1.06 (0.71, 1.58) | 0.79 |
| Model 3 |  | Ref. | 1.05 (0.70, 1.58) | 0.81 |
| Model 4 |  | Ref. | 0.96 (0.59, 1.57) | 0.87 |
| Model 5 |  | Ref. | 0.97 (0.60, 1.58) | 0.91 |
| **Dialysis patients (N=1,770)** | | | | |
| **ICU admitted (N=249)** |  | **Women (N=91)** | **Men (N=158)** | **p-value** |
| **Death, n (%)** |  | **53 (58.2)** | **97 (61.4)** |  |
| Model 1 |  | Ref. | 1.07 (0.77, 1.50) | 0.69 |
| Model 2 |  | Ref. | 1.15 (0.82, 1.61) | 0.41 |
| Model 3 |  | Ref. | 1.18 (0.84, 1.66) | 0.33 |
| Model 4 |  | Ref. | 1.38 (0.94, 2.01) | 0.09 |
| Model 5 |  | Ref. | 1.41 (0.96, 2.07) | 0.08 |
| **Non-ICU admitted (N=1,521)** |  | **Women (N=557)** | **Men (N=964)** | **p-value** |
| **Death, n (%)** |  | **150 (26.9)** | **306 (31.7)** |  |
| Model 1 |  | Ref. | 1.20 (0.99, 1.46) | 0.06 |
| Model 2 |  | Ref. | 1.32 (1.08, 1.60) | 0.01 |
| Model 3 |  | Ref. | 1.32 (1.08, 1.60) | 0.01 |
| Model 4 |  | Ref. | 1.19 (0.96, 1.47) | 0.11 |
| Model 5 |  | Ref. | 1.19 (0.96, 1.46) | 0.12 |

NR: Not reliable (due to fewer events)

Model 1: crude

Model 2: Model 1 + age (continuous), clinical frailty score (continuous)

Model 3: Model 2 + the reason for COVID-19 screening (symptoms-based screening/positive COVID-19 contact or routine screening)

Model 4: Model 3 + smoking (never, current, former), obesity (yes/no), hypertension (yes/no), diabetes (yes/no), heart failure (yes/no), chronic lung disease (yes/no), coronary artery disease (yes/no), and auto-immune disease (yes/no)

Model 5: Model 4 + duration of kidney function replacement therapy (years) and estimated glomerular filtration rate (continuous)

(p-for interaction between sex and ICU admission status in kidney transplant recipients=0.64 and in dialysis patients=0.60)

(ICU admission information missing for 2 kidney transplant recipient and 27 dialysis patients)

**Table S5: Association of sex with 3-month mortality in kidney transplant recipients and dialysis patients with COVID-19 when accounting for country effect* (presented are hazard ratios with 95% confidence intervals)**

| **Transplant recipients (N=1,204)** |  | **Women (N=457)** | **Men (N=747)** | **p-value** |
| --- | --- | --- | --- | --- |
| **Event, n (%)** |  | **85 (18.6)** | **126 (16.9)** |  |
| Model 1 |  | Ref. | 0.88 (0.67, 1.16) | 0.37 |
| Model 2 |  | Ref. | 1.00 (0.75, 1.33) | 0.98 |
| Model 3 |  | Ref. | 0.99 (0.74, 1.31) | 0.92 |
| Model 4 |  | Ref. | 0.88 (0.64, 1.22) | 0.46 |
| Model 5 |  | Ref. | 0.91 (0.66, 1.26) | 0.57 |
| **Dialysis patients (N=3,206)** |  | **Women (N=1,225)** | **Men (N=1,981)** | **p-value** |
| **Event, n (%)** |  | **268 (21.9)** | **536 (27.1)** |  |
| Model 1 |  | Ref. | 1.26 (1.09, 1.46) | 0.002 |
| Model 2 |  | Ref. | 1.46 (1.26, 1.71) | <0.001 |
| Model 3 |  | Ref. | 1.45 (1.25, 1.69) | <0.001 |
| Model 4 |  | Ref. | 1.35 (1.15, 1.59) | <0.001 |
| Model 5 |  | Ref. | 1.36 (1.15, 1.60) | <0.001 |

*A random intercept model was constructed with country as a random factor in a multilevel mixed-effects parametric survival model

Model 1: crude

Model 2: Model 1 + age (continuous), clinical frailty score (continuous)

Model 3: Model 2 + the reason for COVID-19 screening (symptoms-based screening/positive COVID-19 contact or routine screening)

Model 4: Model 3 + smoking (never, current, former), obesity (yes/no), hypertension (yes/no), diabetes (yes/no), heart failure (yes/no), chronic lung disease (yes/no), coronary artery disease (yes/no), and auto-immune disease (yes/no)

Model 5: Model 4 + duration of kidney function replacement therapy (years) and estimated glomerular filtration rate (continuous)

(p-for interaction between sex and type of kidney function replacement therapy=0.02 in fully adjusted model for three month mortality)

**Table S6:** Association of sex and type of kidney function replacement therapy with mortality among patients on kidney function replacement therapy with COVID-19 (Presented are Hazard Ratios with 95% confidence interval)

|  |  | **Female dialysis patients** | **Male dialysis patients** | **Female kidney transplant recipients** | **Male kidney transplant recipients** |
| --- | --- | --- | --- | --- | --- |
| Model 1 |  | Ref. | 1.27 (1.10, 1.47) | 0.81 (0.63, 1.03) | 0.72 (0.59, 0.90) |
| Model 2 |  | Ref. | 1.41 (1.21, 1.63) | 1.71 (1.33, 2.20) | 1.72 (1.37, 21.5) |
| Model 3 |  | Ref. | 1.39 (1.20, 1.62) | 1.61 (1.25, 2.08) | 1.60 (1.28, 2.01) |
| Model 4 |  | Ref. | 1.32 (1.13, 1.55) | 1.69 (1.30, 2.18) | 1.56 (1.24, 1.97) |
| Model 5 |  | Ref. | 1.33 (1.14, 1.56) | 1.99 (1.46, 2.71) | 1.88 (1.41, 2.51) |

Model 1: crude

Model 2: Model 1 + age (continuous), clinical frailty score (continuous)

Model 3: Model 2 + the reason for COVID-19 screening (symptoms-based screening/positive COVID-19 contact or routine screening)

Model 4: Model 3 + smoking (never, current, former), obesity (yes/no), hypertension (yes/no), diabetes (yes/no), heart failure (yes/no), chronic lung disease (yes/no), coronary artery disease (yes/no), and auto-immune disease (yes/no)

Model 5: Model 4 + duration of kidney function replacement therapy (years) and estimated glomerular filtration rate (continuous)

**Figure S1: Flow chart the number of ERACODA participants included in the current analyses**


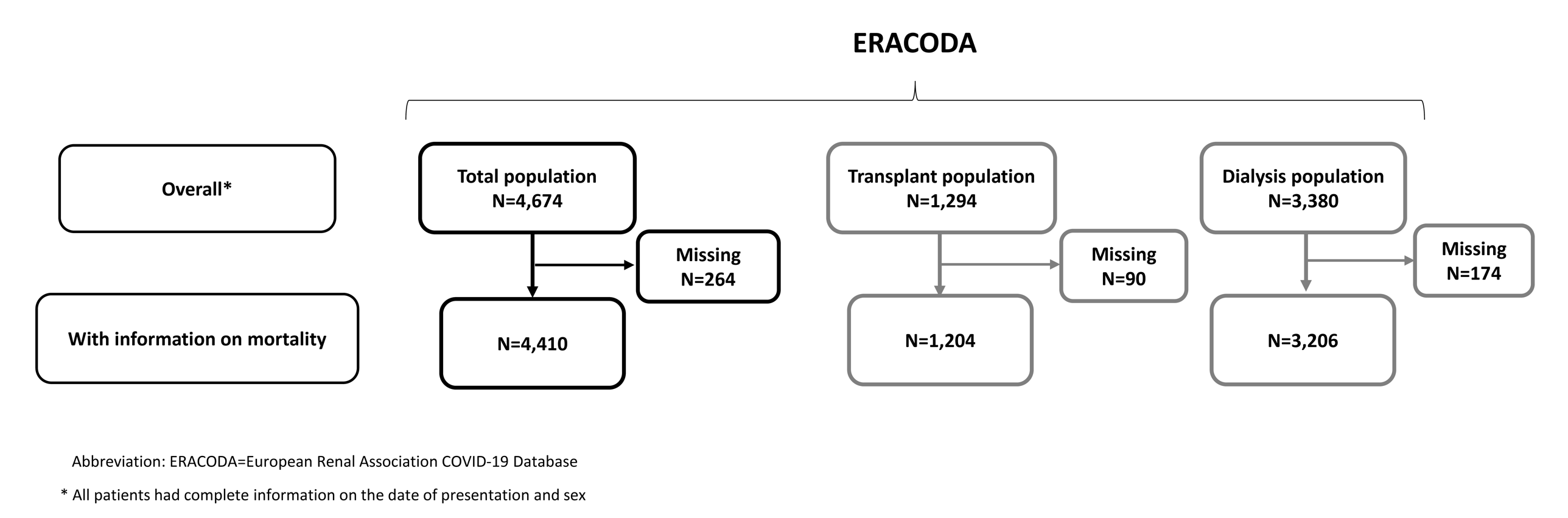


**Figure S2:** **Cumulative incidence of three-month mortality by sex for kidney transplant recipients (Panel A) and Dialysis patients (Panel B)**

**A)**


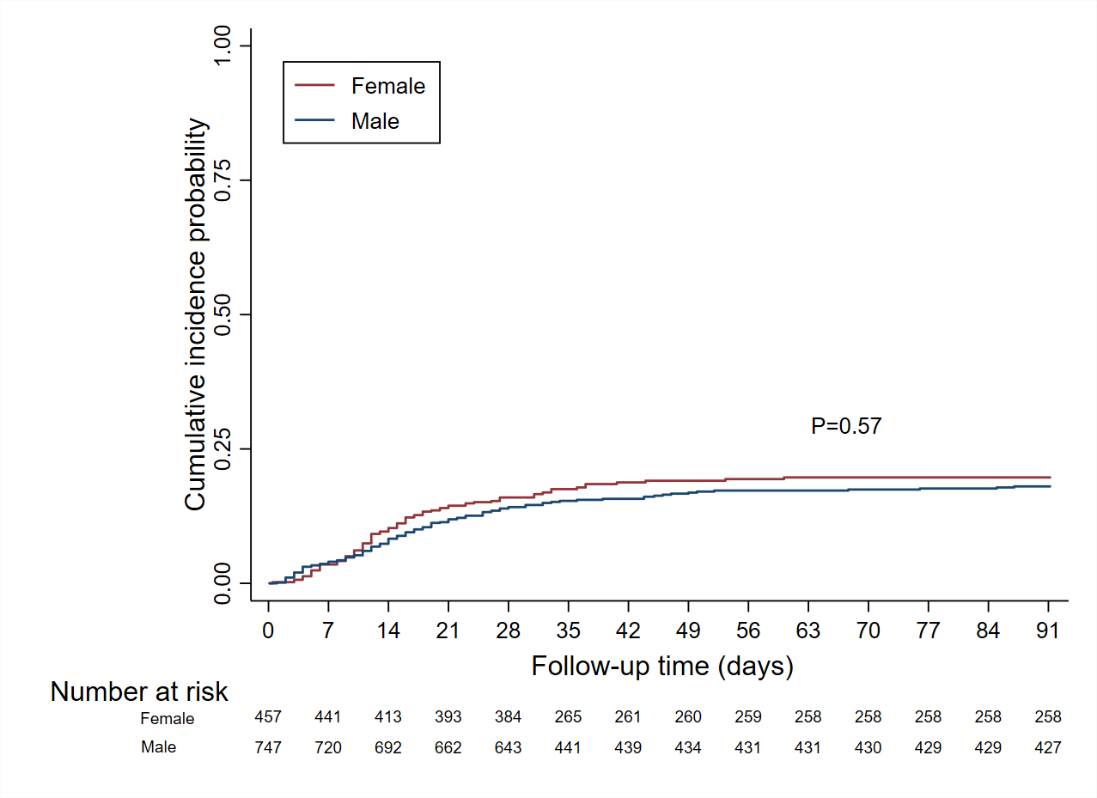


**B)**


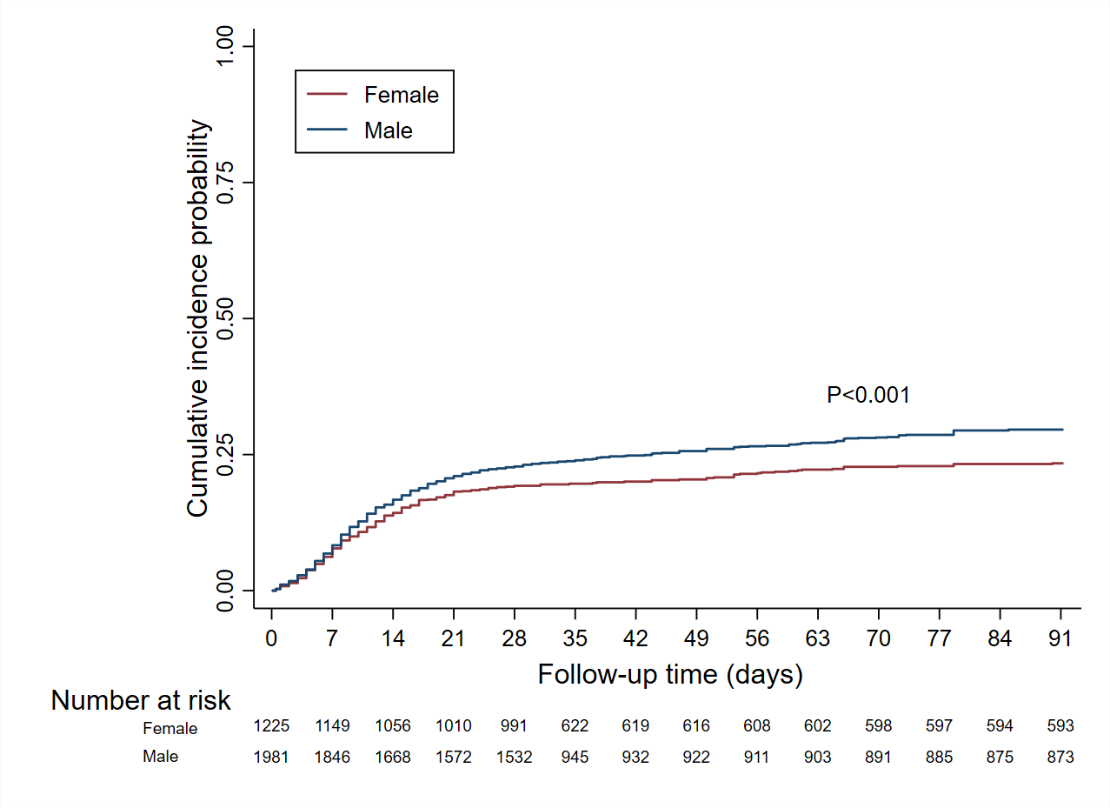

Supplement: Supplementary file 1 — Supplementary Information. [file 41598_2022_22657_MOESM1_ESM.docx]
